# Ewing’s Sarcoma of the Vertebral Body in an Adolescent: A Rare Case Report and Literature Review

**DOI:** 10.7759/cureus.13664

**Published:** 2021-03-03

**Authors:** Teik Chiang Goh, Mohd Yazid Bajuri, Mohamad Fauzlie Yusof, Husna Mohd Apandi, Faris Aiman Sarifulnizam

**Affiliations:** 1 Orthopaedics and Traumatology, Hospital Melaka, Melaka City, MYS; 2 Orthopaedics and Traumatology, Universiti Kebangsaan Malaysia Medical Centre, Kuala Lumpur, MYS

**Keywords:** ewing's sarcoma, primary spine tumour, spinal cord compression, paraplegia

## Abstract

We report the case of a 14-year-old girl who presented with a one-month history of back pain and bilateral lower limb weakness preceded by constitutional symptoms. She neither had a family history of malignancy nor a previous history of trauma. A series of imaging procedures revealed an aggressive lesion of the T12 vertebra with a large soft-tissue component and intraspinal extension leading to spinal cord compression causing cord edema. She underwent urgent posterior instrumentation and fixation of T9 to T12 vertebrae due to worsening neurological deficits. Adjuvant and neoadjuvant chemotherapy with palliative spinal stabilisation were also performed. Features of the lesion were highly consistent with ES on immunohistochemical study and fluorescence in situ hybridization (FISH) analysis for the EWSR1 gene. Postoperatively, both of her lower limbs improved in power and she benefited from regular physiotherapy.

## Introduction

Ewing's Sarcoma (ES) is predominantly seen in the long bones of extremities and commonly involves the femur, pelvis, humerus, tibia, and fibula. The European Intergroup Cooperative Ewing Sarcoma Studies (EI-CESS) concluded that axial skeleton was the major site of occurrence in ES accounting for 54% of cases, followed by 42% in appendicular skeleton and the remaining in other bones [1,2]. Primary malignant sarcomas of the spine are rare accounting for only 3.5% to 14.9% of all primary bone sarcomas [3].

A total of 6% of childhood malignancies arise from primary bone tumours and despite its rarity, ES is the second most common primary bone malignancy led by osteosarcoma [1,2] with 75% comprising of those under the age of 20. As age progresses, a child is less likely to be diagnosed with ES than osteosarcoma. Meanwhile, an increased ES pelvic tumour incidence is reported with increasing age [4]. This condition is not known to be familial or to be a part of any known syndrome with a lack of evidence of environmental aetiologies. However, it was found that Caucasians were more likely to be diagnosed with ES as compared to Africans and East Asians [5].

Patients typically complain of swelling(s) or pain that may be intermittent and progressive within a period of weeks to months which is commonly exacerbated by exertion. Some patients present with an intermittent progression of the condition rather than a steady progression when compared with osteosarcoma. Unfortunately, a few of these malignancies are only identified upon the manifestation of swelling despite going through minor trauma. These swellings may be tender and erythematous with lymphadenopathy appreciated in some patients.

For a lesion involving the spine or sacrum, patients may present with radiculopathy or back pain on top of signs and symptoms of neurological deficit secondary to spinal cord compression which is often delayed and it is uncommon to see rapidly progressing paraplegia. Loss of range of motion of joints can be contributed by juxta-articular lesions while those involving ribcage may have extraosseous masses or pleural extension. Moreover, they may also experience constitutional signs and symptoms such as unintentional weight loss and loss of appetite. Despite many patients presenting with a physically localised disease, the majority are presumed to have subclinical metastases which can be attributed to delayed clinical manifestations or identification of disease. Therefore, a high index of suspicion is necessary for diagnosis predominantly in young patients.

It is said that alleles of the ES gene (EWSR1 gene) are disrupted in the Ewing group of tumours [6]. Translocations affect genes and reassemble to create new fusion genes. This group of tumours has a similar non-random translocation of chromosomes. Thus, they have been regarded as an example for approaching a diagnosis via molecular and cytogenetic techniques. Almost all cases exhibited one of the various translocations: the EWSR1 gene on chromosome 22q12 [6]. Therefore, classifications of bone and soft tissue tumours based on these genetic abnormalities have great potential in approaching diagnosis and management.

## Case presentation

We present the case of a 14-year-old girl with a one-month history of bilateral lower limb weakness and back pain preceded by constitutional symptoms such as unintentional weight loss and loss of appetite. No loss of bowel or bladder control was noted. The patient neither had a family history of malignancies nor a preceding history of trauma. Her immunisations were up to date according to the Malaysian National Immunisation Schedule with normal developmental milestones. Our examination revealed bilateral lower limb weakness with a power of 1/5 to 3/5. Furthermore, hyperreflexia of bilateral lower limbs, clonus, and reduced sensation from L3 to S5 bilaterally were also noted.

**Figure 1:**
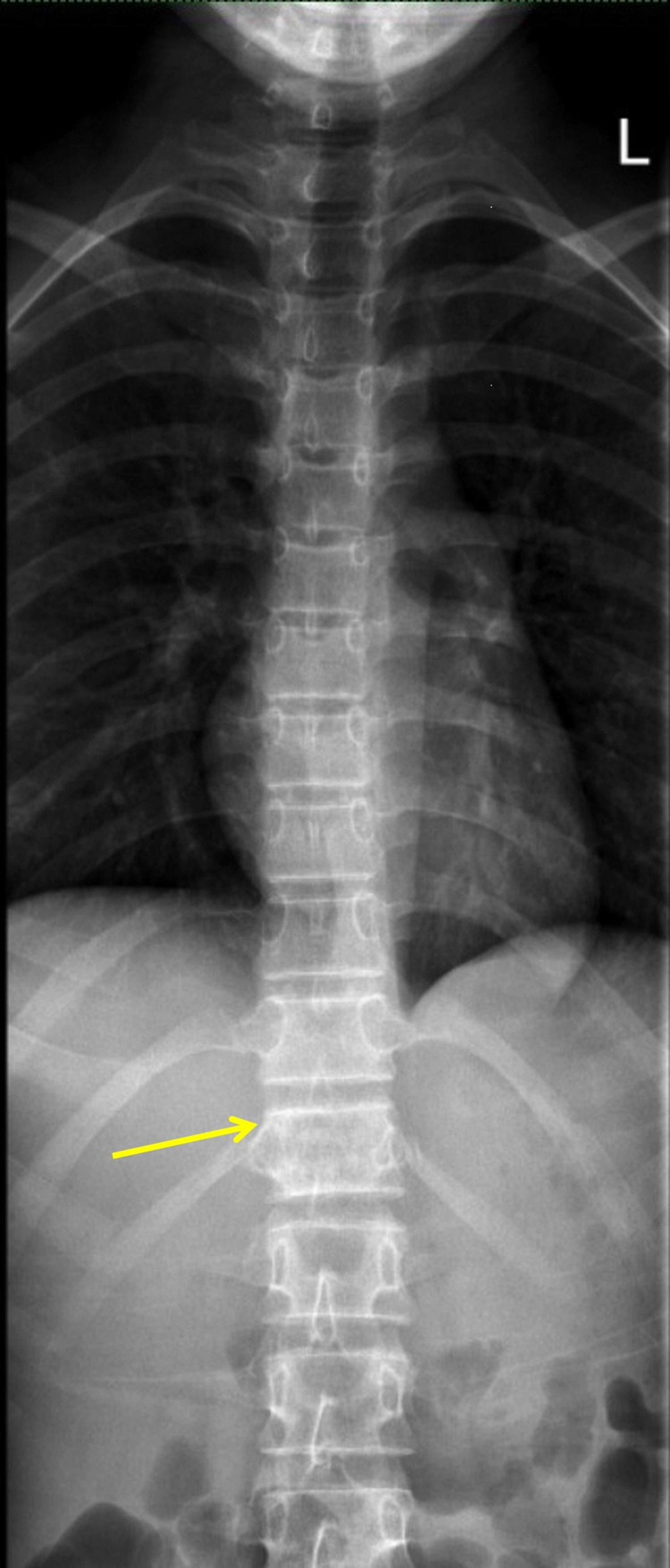
Pre-operative plain radiograph of the spine (anteroposterior view). The arrow shows a mixed sclerotic bony lesion of the T12 vertebral body with preserved vertebral height.

**Figure 2:**
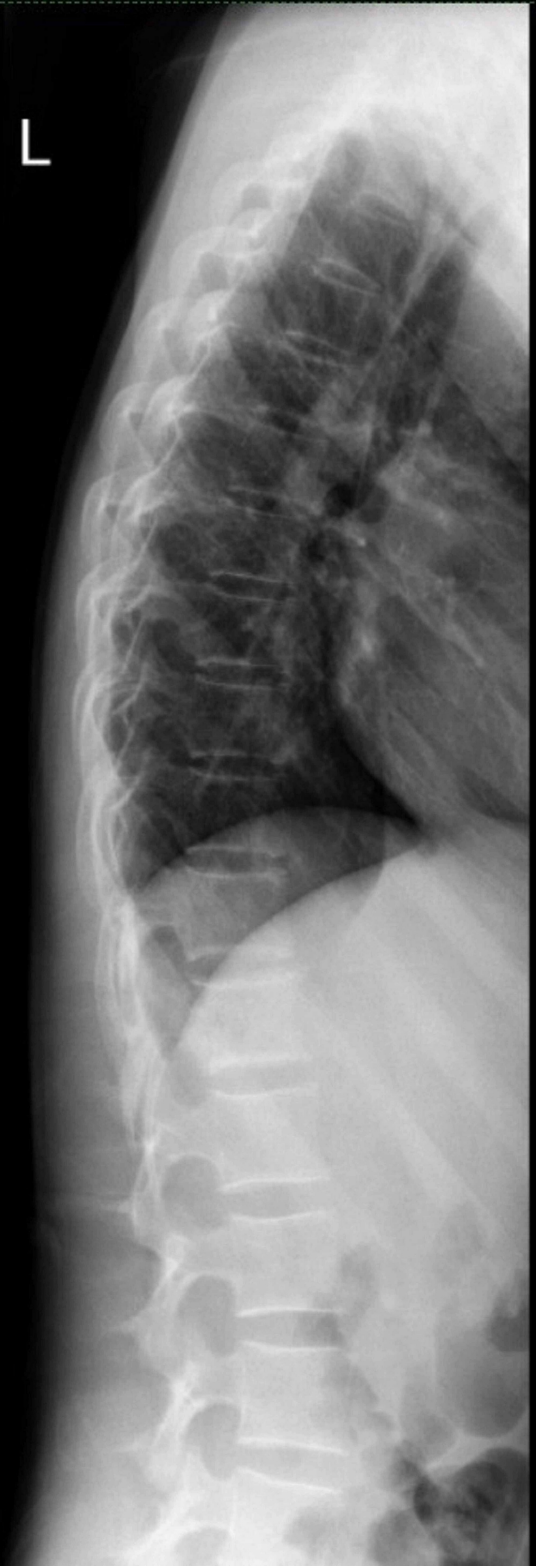
Pre-operative plain radiograph of spine (lateral view).

**Figure 3:**
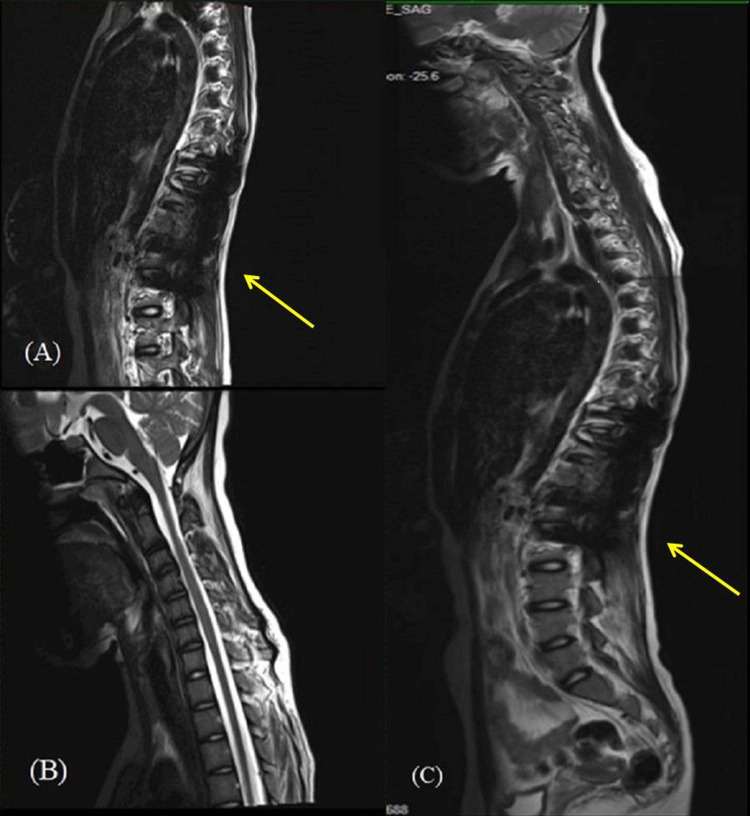
Pre-operative MRI of an aggressive lesion of the T12 vertebra (arrow); the large soft-tissue component and intraspinal extension led to spinal cord compression, resulting in cord edema.

A series of imaging procedures were performed beginning with a plain radiograph of the spine which showed a mixed sclerotic bony lesion of the T12 vertebral body with preserved vertebral height (Figures 1, 2). No fractures were seen in the visualised bones and no lesions were revealed in the other visualised spines. An MRI revealed an aggressive lesion of the T12 vertebra with a large soft-tissue component and intraspinal extension that led to spinal cord compression, resulting in cord edema (Figures 3A-3C). A computed tomography scan of the thorax, abdomen, and pelvis (CT TAP) also revealed an aggressive T12 vertebral bony lesion with a soft-tissue component likely representing a malignancy with the presence of subcentimeter para-aortic nodes. A bone scan was done and the result revealed the absence of distant metastasis. 

The patient already had a neurological deficit but unfortunately, her condition worsened and therefore, a CT-guided biopsy was done to further identify the nature of the lesion. Then, she underwent urgent surgical intervention in the form of posterior instrumentation and fixation from T9 to T12 spinal vertebrae. Intraoperative findings showed a right paravertebral lesion extending from the level of T11 to L1 causing cord compression. Post-operatively, her lower limb power improved from 1-3/5 to 3-4/5. She was able to stand with a walking frame and benefited from regular physiotherapy.

A follow-up MRI showed that the T12 vertebral mass had undergone no significant change from previous imaging. A positron-emission tomography (PET) scan, after a cycle of vincristine, ifosfamide, doxorubicin, etoposide (VIDE), showed a smaller residual paravertebral lesion at T12, with a hypermetabolic lesion at the superior segment of the left lower lobe raising suspicion of a metastatic nodule.

**Figure 4:**
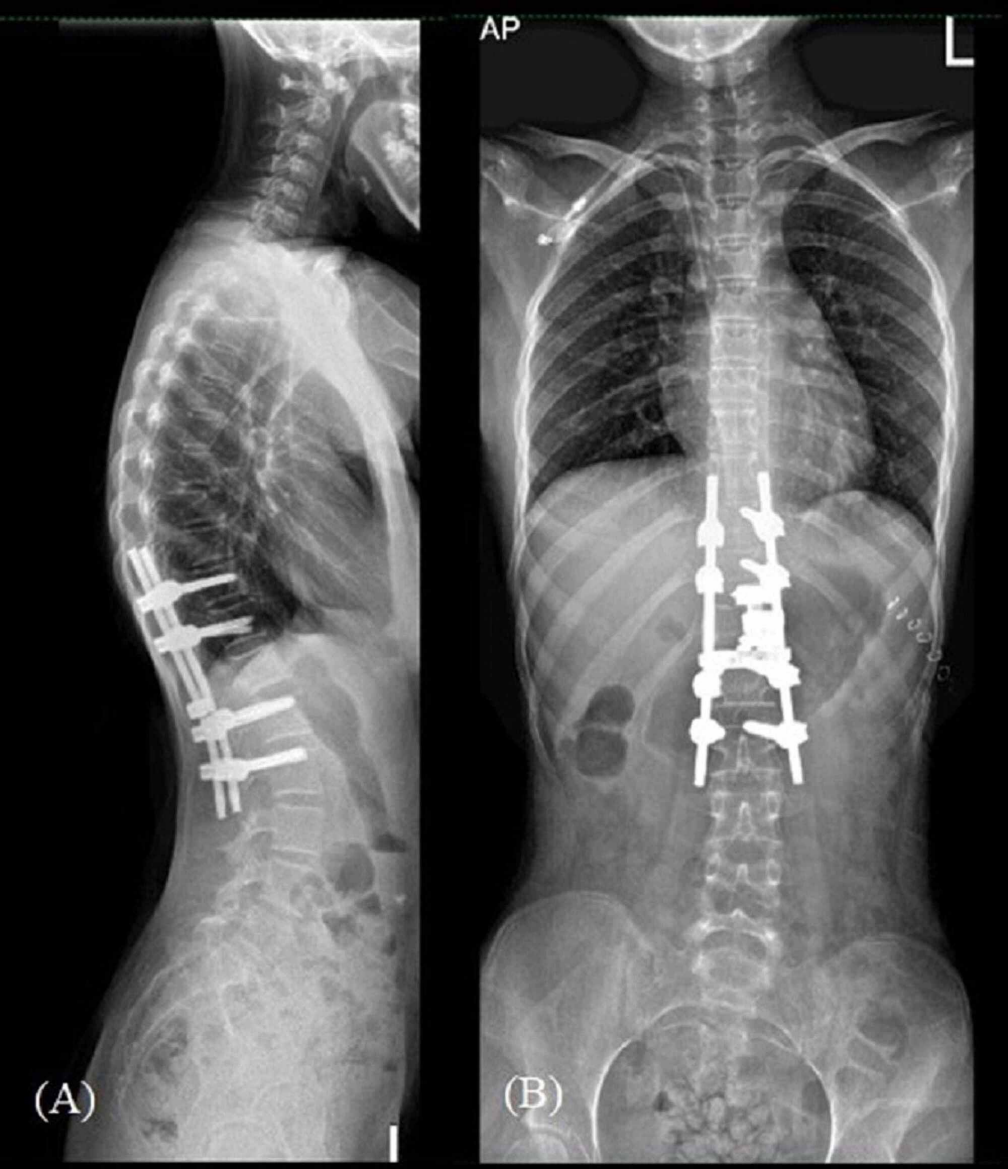
Immediate radiograph post definitive vertebrectomy and spinal fusion.

Immunohistochemical studies were suggestive of malignant cells and fluorescence in situ hybridization (FISH) analysis for the EWSR1 gene showed the presence of EWSR1 gene rearrangement which was highly consistent with ES. Post chemotherapy, the patient underwent a definitive vertebrectomy and spinal fusion; the immediate postoperative radiographs are shown in Figures 4A, 4B. She subsequently benefited from six cycles of neoadjuvant chemotherapy (VIDE). Postoperatively, her bilateral lower limbs’ power improved from 3/5 to 4/5.

## Discussion

ES is the second most common bone neoplasm in the paediatric population with a male-to-female ratio of 1.6:1 [7]. The highest incidence occurred in the second decade of life as seen in our patient and typically involves the long bones of extremities and pelvis, presenting predominantly with swelling and pain. Primary vertebral Ewing's sarcoma has been classified into sacral and non-sacral subtypes on the differences in treatment response and survival rate. Non-sacral type constitutes approximately 0.9% of all cases. Patients mostly presented with low back pain followed by palpable swelling(s). Furthermore, patients may present late with neurological deficits due to spinal cord compression. For our patient, she presented with worsening bilateral lower limb weakness preceded by constitutional symptoms.

ES often tends to invade the spinal canal from the paravertebral soft tissue component through the intervertebral foramen, compressing the cord circumferentially. Therefore, laminectomy is an effective modality for cord decompression. Thorough investigations such as haematological parameters and appropriate imaging are needed to complement the clinical history and physical examinations to arrive at a proper diagnosis. Multiple imaging modalities can be used to identify and further define the lesions. A plain radiograph should be taken first to identify the presence of destructive lesions with poor circumscription which have been described to have a “moth-eaten” appearance. ES is also known to have Codman’s Triangle appearance which usually represents an aggressive bone lesion apart from an “onion-peel” appearance due to periosteal reaction [8]. CT scan is typically necessary to ensure a better delineation of actual damage to the cortices whereas MRI is superior in detailing tumour sizes and extension [9]. Unfortunately, approximately 10% to 15% of patients are known to suffer from pathological fractures during diagnosis [8].

Apart from that the above, identifying the metastases aids in determining the management protocol for which a CT TAP scan is warranted. To exclude the presence of skip lesions (i.e. medullary disease within the same bone but not in direct contiguity with the primary lesion), imaging of the entire involved bone is required. Appropriate pre-staging workup including a bone scan may be necessary prior to the institution of therapy and for further monitoring because ES is usually a multifocal disease. Biopsy of the primary tumour is beneficial especially if other investigations are inconclusive. However, it should be considered carefully to avoid tumour disruption or spillage due to its invasive nature which may necessitate the expertise of an interventional radiology unit. Biopsies can be of immense value as they can be both diagnostic and therapeutic. About 90% to 98% of biopsies have been known to turn out a tissue-based diagnosis [10].

The options for management of ES depend on the extent of the disease, whether local or metastatic, and consist of chemotherapy, radiotherapy, and surgical intervention. Treatments for a local disease include both adjuvant and neoadjuvant chemotherapy which can be followed by targeted treatment leading to tumour size reduction which aids in subsequent surgical resection [11]. According to the National Comprehensive Cancer Network (NCCN) guidelines, there are various courses of treatment including preoperative radiotherapy or chemotherapy which can be supplemented by postoperative radiotherapy [12]. Chemotherapy is available with induction and neoadjuvant agents, in addition to as postoperative treatment. Europeans recommend administration of vincristine, ifosfamide, doxorubicin, etoposide (VIDE) whereas Americans have been known to practice administration of both neoadjuvant and adjuvant chemotherapy using vincristine, doxorubicin, and cyclophosphamide (VDC), with alternating cycles of ifosfamide and etoposide (IE). A randomized phase III clinical trial (IESS-III study) showed that in a localised illness, 69% of patients were found to have an improved five-year relapse-free survival rate when given IE in addition to VDCA (vincristine, doxorubicin, cyclophosphamide, dactinomycin) as opposed to only 54% of patients such survival in the VDCA only group [13].

Advances in the medical field have dramatically improved treatment regimes. According to Delattre O et al., the five-year survival rate has risen from 59% to 78% in children less than 15 years old, whereas the percentage in adolescents between 15 to 19 years old has tripled from 20% to 60% [14]. Factors for monitoring the efficacy of the treatment include alleviation of pain, improvement of haematological parameters (lactate dehydrogenase) and radiographic imaging, and the presence of necrotic tissue in surgically removed specimens. Despite having the ability to reduce or control tumour size, cautious use of radiotherapy in treating ES is necessary to not cause more harm to the patient. Meticulous care should be instituted to ensure that the risks do not outweigh the benefits for patients due to probable radiation-induced malignant transformation. Many patients have also been known to already progress into the metastatic stage of malignancy due to late presentation and diagnosis. Multimodal treatment is usually recommended as relapse rates are about 80% to 90% in patients that undergo local therapy as a stand-alone treatment [15].

Surgical intervention is not often offered to patients who have vertebral involvement due to the complexity of the resection. It may be challenging to obtain negative margins without compromising functionality [16]. However, in some cases, a palliative stabilisation of the spine for spinal cord and adjacent structure protection alongside sufficient load distribution across the spine is warranted. The spinal instability neoplastic scoring (SINS) may be used to identify the need for spinal stabilisation consisting of six factors namely location of the lesion, pain characteristics, nature of the bony lesion, spinal alignment according to radiographic findings, degree of destruction towards the vertebral body, and posterolateral involvement of spinal elements. These factors have individual scores and their sum will determine the degree of spinal stability. The score ranges from 0 to 18 in which a score below 6 is defined as a stable spine, a score between 7 and 12 may represent indeterminate or doubtful stability whereas a score of 13 to 18 signifies an unstable spine. In many cases, a patient with a score of ≥8 is highly recommended to undergo surgical intervention for spine stabilisation. Due to current advancements in surgical techniques and equipment, interventions can be done promptly with minimal side effects [17].

Continuous monitoring of the patient’s well-being and progress is pertinent once treatment has been instituted. The consensus-based guidelines from National Comprehensive Cancer Network (NCCN) and Children’s Oncology Group (COG) have outlined recommendations for post-treatment surveillance which include three-monthly monitoring for the first two years, followed by six-monthly monitoring between the third year and fifth year and annual monitoring subsequently. Parameters monitored during these visits should include a full blood count, imaging of the primary site and chest which are usually done via CT scan. There are no solid guidelines pertaining to the duration of follow-ups due to probable relapse in later stages, thus lifelong follow-up is recommended [16,17]. According to Grubb et al., those who are responsible for post-treatment surveillance should be concerned about radiation exposure and the risk of secondary malignancies, especially in younger patients. They found that there was a 33% 5-year survival rate seen in their patients. Regardless, a compelling interconnection could not be identified between the location of the spinal tumour and multiple factors including the duration of being disease-free, overall survival, and incidence of metastasis [18].

## Conclusions

ES of the vertebral body is a rare occurrence that may be seen in the paediatric or adolescent population. A thorough workup is crucial to arrive at a definitive diagnosis. A good clinical acumen is of utmost importance in ensuring prompt diagnosis and intervention for a better prognosis.
